# Change Engagement, Change Resources, and Change Demands: A Model for Positive Employee Orientations to Organizational Change

**DOI:** 10.3389/fpsyg.2020.531944

**Published:** 2020-11-06

**Authors:** Simon L. Albrecht, Sean Connaughton, Kathryn Foster, Sarah Furlong, Chua Jim Leon Yeow

**Affiliations:** ^1^School of Psychology, Deakin University, Geelong, VIC, Australia; ^2^Nous Group, Melbourne, VIC, Australia; ^3^Portable, Melbourne, VIC, Australia; ^4^Ernst and Young Advisory Pte. Ltd., Singapore, Singapore

**Keywords:** change engagement, change engagement model, change-related organizational resources, change-related job resources, change-related job demands

## Abstract

The purpose of this conceptual article is to introduce the construct of change engagement and a model that also consists of change-related organizational resources, change-related job resources and demands, and change-related personal resources. We propose that change engagement is a construct that is theoretically and practically useful for understanding employee reactions to and adoption of organizational change. Drawing from existing models of employee engagement, we add to the change literature by identifying salient change-related organizational resources, job resources, job demands, and personal resources in a previously validated framework that brings together the literature on both engagement and change. By using the proposed change engagement framework, practitioners and researchers will potentially be able to effectively diagnose, manage, and optimize employee change readiness and enthusiasm for ongoing change. Furthermore, the change engagement model (CEM) provides practitioners and researchers with a comprehensive and practically useful model that will be easy to comprehend and communicate. The model can be applied to the planning, implementation, and evaluation of discrete change initiatives, as well as to ongoing change. The model is therefore well-suited to contemporary organizational contexts where change is widely recognized to be a constant.

## Introduction

Since Lewin’s (1946, 1947, 1951) seminal work identifying unfreeze, move, and refreeze as core phases of successful organizational and community change, researchers and practitioners have been evolving principles and practices for planning, implementing, and evaluating episodic or discrete organizational change. Although much progress has been made in understanding organizational change, there have been recent calls for a fundamental rethink about the foundational beliefs that underpin the way that organizational change is understood (e.g., Jick and Sturtevant, 2017). In contrast to earlier analyses of planned organizational change, organizational change is now widely recognized as a constant in contemporary organizational contexts (By, 2005; De Meuse et al., 2010; Tsaousis and Vakola, 2018). As a consequence, practitioner commentary and research attention continue to be focused on understanding how best to manage change in volatile, uncertain, ambiguous, and complex environments (e.g., Armenakis and Harris, 2009; Oreg et al., 2013; Bennett and Lemoine, 2014).

Employee attitudes to change have consistently been shown to have a significant effect on the success of change initiatives. It has been argued that change can only be managed successfully to the extent that employees adopt new processes and ways of working by changing the way they think, feel, and behave (Piderit, 2000; Choi, 2011; Oreg et al., 2011). Contemporary organizations therefore face the challenge of understanding and managing how organizational, job, and individual difference factors contribute to the formation of positive employee attitudes to change (Caldwell, 2013). In this article, we propose that “change engagement” is a useful construct for understanding and operationalizing positive employee attitudes to change. We also propose a change engagement model (CEM) that explains the relationships between change-related organizational resources, change-related job resources and demands, change-related personal resources, and change engagement and how they impact on change-related outcomes. After first overviewing the literature on attitudes to change, we elaborate on the construct of change engagement and the CEM.

Attitudes to change refer to employees’ overall positive, negative, or neutral thoughts; feelings; and behavioral intentions regarding change initiatives proposed or implemented by their organization (Lines, 2005). Negative attitudes have been variously defined and operationalized by constructs such as resistance to change (e.g., Ford et al., 2008; Jones and Van de Ven, 2016) and pessimism and cynicism about organizational change (e.g., Andersson and Bateman, 1997; Abraham, 2000; Wanous et al., 2000). Positive attitudes to change have been defined and operationalized by constructs such as acceptance of change (Iverson, 1996), openness to change (Wanberg and Banas, 2000), change readiness (Armenakis et al., 1999; Holt et al., 2007), and affective commitment to change (Herscovitch and Meyer, 2002).

Both positive and negative attitudes to change have been shown to be associated with important employee attitudinal, behavioral, and performance outcomes. Thundiyil et al. (2015) meta-analysis showed negative attitudes such as change cynicism are negatively associated with job satisfaction, affective commitment, and organizational citizenship behavior. Positive attitudes such as openness to change have been shown to be positively associated with positive behavioral and attitudinal outcomes such as system usage, job satisfaction, affective commitment, and turnover intentions (e.g., Wanberg and Banas, 2000; Chawla and Kelloway, 2004; Augustsson et al., 2017). It is noteworthy that most of the research on attitudes to change has focused on understanding the factors that influence employees’ attitudes to planned and discrete change initiatives (e.g., van den Heuvel et al., 2017), and less research has been focused on understanding the factors that drive success in constantly changing work environments (Pluta and Rudawska, 2016).

A number of researchers (e.g., Piderit, 2000; By, 2005; Elias, 2009; Mathews and Linski, 2016) have argued that rather than focusing on negative attitudes such as change resistance or change cynicism, it is more important to understand positively oriented employee attitudes to change. Mathews and Linski (2016), for example, argued that “the negative and deficiency-based approach used to frame the subject of employee resistance to change seems counterproductive to the end goal of learning how to positively address resistance and implement change successfully” (p. 963). Similarly, Bouckenooghe (2010) recognized that a positive versus a negative focus on change will more likely be associated with “seizing opportunities for improvement, motivating people to perform at a higher level, and …creating commitment to change” (p. 508). As described below, just as employee engagement is a positive motivational construct (Schaufeli, 2013; Youssef-Morgan and Bockorny, 2013), the proposed construct of change engagement and the proposed CEM focus mostly on positive employee experiences of change.

In order for organizational change to be implemented efficiently and successfully, beyond merely being open and receptive toward change (Miller et al., 1994), employees need to be willing to positively and actively engage in change processes. This is because ongoing successful organizational change requires employees who feel energized by change, who are willing to experiment with change, and who actively support and adopt proposed new initiatives through changes in their attitudes and behavior (Armenakis et al., 1993; van Emmerik et al., 2009). Within the engagement literature (e.g., Bakker et al., 2011; Bakker and Demerouti, 2014), and drawing from well-established theoretical models of job-related affective well-being (e.g., Warr, 1990; Russell, 2003), engagement is considered to be a more affectively activated and higher arousal construct than either job satisfaction or commitment (e.g., Albrecht, 2010; Inceoglu and Fleck, 2010). In parallel, “change engagement” potentially provides a distinct and more proactive and agentic (Ghitulescu, 2006; Gawke et al., 2019), high-arousal (Armenakis and Harris, 2009), and motivational (Elias, 2009) expression and extension of previously researched positive change attitudes such as readiness for change, openness to change, or commitment to change. Gawke et al. (2019), for example, validated a measure of agentic behaviors that, in part, assesses employee initiative in effecting organizational change. Ghitulescu (2006) reviewed research findings that highlight the critical importance of proactive and active change-oriented attitudes and behavior for enacting successful organizational change.

**FIGURE 1:**
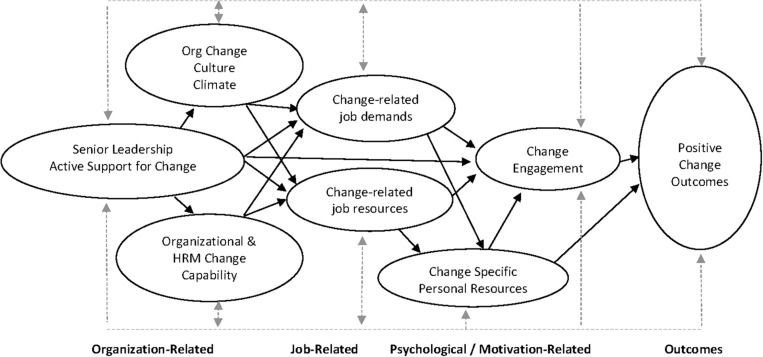
Change engagement model.

After briefly overviewing employee engagement as a construct and reviewing its underpinning theory, the notion of change engagement will be further described and contextualized within a change model focused on change engagement, change resources, and change demands. In so doing, this article aims to provide integration across the change management and engagement literatures that, until now, have largely run independently. In line with well-researched and well-established relationships in the engagement literature, Figure 1 shows how organization-related change resources, job-related change resources, job-related change demands, and personal-related change resources lead to change engagement and subsequently to downstream attitude, behavioral, and performance outcomes. The dotted lines suggest that all proposed relationships in the model can be direct, indirect, and reciprocal. Table 1 shows a number of, for example, change-related organizational resources, job resources, job demands, personal resources, and change-related outcomes.

**TABLE 1:** Example elements of change-related organizational resources, job resources, job demands, change engagement and change outcomes.

Change-related organizational resources			Change-related job resources
Senior leadership	HRM and organizational change capability	Organizational culture and climate	
• Clear communication about importance & constancy of change • Active & visible sponsorship, support & resourcing for ongoing change • Clarifying outcomes and behavioral expectations for change • Transformational change leadership: - Inspiring and involving others - Modeling the way	• Dedicated change resources and infrastructure for change capability • Integrated and strategically aligned HR and OD change resources • Change focused HR & OD functions: selection, socialization, performance management, training & development • Change portfolio and change capacity management	• A culture for change with explicit change-related values. • A climate for change where change-related policies, practices and procedures are shared and supported • Empowerment and team orientation with external and customer focus	• Change autonomy • Change participation and involvement • Supervisor support for change • Information about ongoing change • Ongoing training and development about change • Skill development opportunities in change • Feedback about ongoing change

**Change-Related Job Demands**	**Change-Related Personal Resources**	**Change Engagement**	**Change Outcomes**

• Change-related work overload • Change-related job ambiguity • Change-related role conflict • Change-related job insecurity and uncertainty • Change-related emotional demands • Change-related work intensification	• Change-related PsyCap: hope, optimism, resilience & self-efficacy • Change-related organization based self esteem • Change-related meaning-making • Change-related mind-sets • Change-related self-management & self-leadership • Change-related psychological safety • Felt obligation for constructive change • [Personality/dispositional factors]	• Enthusiasm for change • Involved & participating in change • Focused energy for change • Willingness to actively support change • Striving for change success	• Successful implementation and adoption of ongoing change • Return on change investment • Innovation
			

## Employee Engagement and the Job Demands–Resources Model

Employee engagement has remained a “hot topic” (Macey and Schneider, 2008) over the past 20 years because enthusiastic, motivated, and involved employees have been recognized as a critical source of competitive advantage (Albrecht et al., 2015; Shuck et al., 2017; Schneider et al., 2018). Engaged employees feel positive and are involved in their work and willing to work toward the achievement of work role and organizational goals (Macey et al., 2009; Albrecht et al., 2015).

The job demands–resources (JD-R) model (Bakker and Demerouti, 2007, 2008, 2018) is a widely used and widely cited theoretical explanation of engagement (Bakker and Demerouti, 2014). The JD-R describes how job resources (e.g., autonomy, feedback, and supervisor support) and personal resources (e.g., self-efficacy, optimism, and resilience) directly influence work engagement via a positive motivational pathway by providing employees with what they need to complete their work. Job demands (e.g., role conflict, role ambiguity, and emotional demands), on the other hand, are proposed to deplete energy and directly influence negative employee outcomes such as strain and burnout. Numerous cross-sectional, meta-analytic, and multilevel studies (e.g., Crawford et al., 2010; Halbesleben, 2010; Christian et al., 2011; Bakker and Demerouti, 2018) have supported the relationships proposed by JD-R models. Recent extensions of the JD-R have proposed that beyond consideration of job resources, personal resources, and job demands, it is also important to take account of organizational resources such as clarity of organizational vision, human resources management (HRM) systems, strategic alignment, and organizational climate (e.g., Barrick et al., 2015; Albrecht et al., 2018).

Although it has been argued that the JD-R is particularly useful when examining changing working contexts and the changing nature of work (Bakker and Demerouti, 2014), only a limited amount of empirical research has applied the JD-R to understanding the relationships between job resources, job demands, and employee evaluations of change (e.g., van Emmerik et al., 2009; Lee et al., 2017; Bellou et al., 2018). It is here argued that the model can serve to organize theoretical propositions and the wide range of constructs previously examined within the change literature (e.g., change demands, change resources, coping resources, and personal resources) into a coherent and practically useful framework that can help explain motivational constructs such as employee change readiness. Lee et al. (2017), for example, using the JD-R as a framework, conceptualized organizational change environments in terms of job demands and resources as determinants of attitudes toward change. Although Lee et al. (2017) examined only a limited number of demands and resources, the use of the model helped address what has been acknowledged as a lack of theoretical underpinning and integration in the research on organizational change (Oreg et al., 2011; Straatmann et al., 2016).

As with the limited number of researchers who have focused on the relationships between job resources, job demands, and employee evaluations of change (e.g., Michel and Gonzáles-Morales, 2013), only a limited number of researchers have investigated associations between engagement and attitudes to change (e.g., Marinova et al., 2015; van den Heuvel et al., 2017; Matthysen and Harris, 2018). Marinova et al. (2015), for example, suggested that employees who experience higher levels of engagement are more likely to take an active role in change efforts and generally be more positive toward change. Marinova et al. (2015) also provided evidence to show that engagement mediates the relationship between job characteristics (e.g., autonomy, task significance, and job complexity) and change-oriented behavior. The present article aims to extend on JD-R literature and identify change-related organizational resources, job resources, job demands, and personal resources that are likely to be associated with employee change engagement as an important attitudinal and motivational construct.

## Change Engagement

As previously noted, although the engagement literature and the change literatures have largely progressed along independent lines (van den Heuvel et al., 2010), a limited number of practitioners and researchers have indirectly referred to the notion of employee engagement in change. Miller (2011), for example, identified “powerful engagement processes” as a “critical success factor” for effective organizational change. Dhensa-Kahlon et al. (2015) proposed that positive emotional experiences at work will be associated with increased “engagement in change.” Similarly, Straatmann et al. (2016) argued that high levels of employee engagement in change are essential to successful organizational change. Straatmann et al. (2016) operationalized engagement in change as a willingness to enact behaviors consistent with the implementation of a specific change. “Willingness” and “intention,” as constructs, are fundamental to attitude theories such the theory of reasoned action and the theory of planned behavior (e.g., Ajzen and Fishbein, 1980; Ajzen, 1991). To date, however, a more generic and widely applicable conceptualization of “change engagement,” as an analog of employee engagement, has not been explicitly defined within the change literature. The construct of change engagement potentially provides a more energized and motivational expression of positive change-related attitudes than constructs such as openness to change, readiness for change, commitment to change, and willingness to engage in organizational change.

Extrapolating from existing definitions of engagement (González-Romá et al., 2006; Macey et al., 2009; Albrecht, 2010), change engagement is here defined as an enduring and positive work-related psychological state characterized by a genuine enthusiasm and willingness to support, adopt, and promote organizational change. The definition captures the essential qualities of positive energy, involvement, and focused effort that characterize employee engagement (González-Romá et al., 2006; Macey et al., 2009), but applies them to the context of change. As previously noted, change engagement has a more agentic quality, experienced as enthusiasm, energy, involvement, and vigor, as opposed to alternative constructs such as change commitment and openness to change. Although a limited number of researchers have also recently suggested change attitude items that are imbued with more positive affect (e.g., Tsaousis and Vakola, 2018; Rafferty and Minbashian, 2019), none have done so by explicitly addressing the construct of change engagement embedded within a theoretically derived nomological framework or change model. Example change engagement items might include the following: “I am enthusiastic about change in this organization,” “I feel energized when we are going through change,” “I am willing to invest my time and energy to the implementation of organizational change,” and “I am willing to convince colleagues of the benefits of ongoing change.”

## Change Engagement, Change Resources, and Change Demands—A Model

The CEM, as shown in Figure 1, draws from the JD-R to provide a theoretically grounded framework to understand positive employee responses to change. Consistent with Pettigrew et al. (2001) call for organizational change theories to have both scholarly rigor and practical relevance, the model is offered as an encompassing and comprehensive model of organizational change that integrates many divergent lines of existing change research into a coherent, flexible, and practically useful model. Just as the JD-R, in its extended forms, has proved helpful to organizations wanting to understand the antecedents and outcomes of engagement, it is here proposed that change-related resources and demands can be helpful in conceptualizing the important antecedents or preconditions for change engagement. Analogous to well-established JD-R evidence about how different categorizations of resources and demands influence engagement, Figure 1 proposes that change-related organizational resources, job resources, job demands, and personal resources can potentially directly and indirectly influence change engagement and positive organizational change outcomes.

## Change-Related Organizational Resources

Organizational resources are system-level aspects of the organizational environment that are not role specific and that both directly and indirectly influence employee attitudes and behavior (Albrecht et al., 2018). In a change context, organizational resources broadly include senior leadership’s active support of change, HRM change systems and supports, organizational change capability, and organizational change culture and climate (Albrecht et al., 2018). As shown in Figure 1, organizational change resources can directly and indirectly influence employee experiences of change-related job and personal resources, change-related job demands, change engagement, and positive change outcomes. As discussed below, and as shown in Table 1, the existing literature identifies a number of important elements for each of the broad categories of organizational change resources. The non-exhaustive list of elements in Table 1 is drawn from both the engagement literature (e.g., Albrecht et al., 2015; Barrick et al., 2015) and organizational change literature (e.g., Rafferty et al., 2013; Jick and Sturtevant, 2017; Vakola and Petrou, 2018). A number of change-related organizational resources are described below.

Senior leadership’s active support of change is recognized as fundamental to the success of any particular change initiative and for successful ongoing change (Kotter, 1990; Armenakis and Bedeian, 1999). Resources that senior leadership can provide to support successful change include clear change purpose and vision, clear communication of information about change, and active sponsorship and support for change (Rafferty et al., 2013; ten Have et al., 2017; Albrecht et al., 2018).

In addition to senior leadership’s active support of change, organizational and HRM change capabilities are increasingly being recognized as essential to the management and success of ongoing organizational change (Trahms et al., 2013; Costanza et al., 2016). Costanza et al. (2016), for example, noted that in order “to respond to environmental threats and opportunities, organizations must develop an infrastructure and capabilities that allow adaptation to such environmental changes and, ultimately, survival” (p. 361). As such, organizations need well-developed and interrelated systems that allow them to function, adapt, and be “macro-organizationally ready” (Vakola, 2013) in the face of ongoing change. Specific human resources and organizational development functions that need to be in place to support, promote, and embed ongoing change capability include change-focused selection, socialization, performance management, and training and development (Shipton et al., 2006; Fugate, 2012).

Schneider et al. (1996) noted that “what people in an organization experience as the climate and believe is the culture ultimately determines whether sustained change is accomplished” (p. 18). Although culture and climate are often conceptualized as global or “molar” constructs, just as researchers have focused on a culture or climate of fairness (Colquitt et al., 2002), engagement (Albrecht, 2014), and innovation or adaptability (e.g., Costanza et al., 2016), it also makes conceptual sense to focus on a “culture or climate for change.” For present purposes, organizational change culture refers to espoused and experienced organizational values (Schein, 1990; Argyris, 1993) that are relevant to ongoing change. Such values include innovation, creativity, intrapreneurship, flexibility, responsiveness, nimbleness, and adaptability (e.g., Brown and Leigh, 1996; Denison et al., 2014; Gawke et al., 2019). Change climate could refer to employee perceptions about whether their organization’s policies, practices, procedures, and expected behaviors are supportive of, and promote, organizational change (Schneider et al., 1996).

Overall, there is considerable research evidence showing that the organizational resources and the organizational context are critically important to organizational success, particularly in constantly changing organization environments (Armenakis and Harris, 2009). Figure 1 proposes that when senior leaders actively promote, sponsor, resource, and support ongoing organizational change, they will directly or indirectly influence an organization’s change capability, culture and climate for change, individual employee change resources, and change engagement. Table 1 shows a non-exhaustive list of change-related organizational resources that have been shown to contribute to successful ongoing organizational change.

## Job-Related Change Resources

As previously noted, the willingness of individual employees to change the way they think, feel, and behave, to a very large extent, determines the success of organizational change initiatives (Armenakis et al., 1993; By, 2005). Meta-analyses have shown that job-level employee experiences have an important influence on a wide range of attitudinal, behavioral, and performance outcomes (Morgeson and Humphrey, 2006; Crawford et al., 2010; Christian et al., 2011). It is therefore important to be able to identify change-related job-level experiences that can influence employee attitudes to change. Change-related job resources are here defined as the psychological, physical, technological, informational, financial, and social supports; arrangements; and supplies perceived by employees as available to help them successfully adopt and adapt to the organizational changes that impact their job role.

It has been well-established by change researchers that job-level resources such information about change, participation in change, supervisor support for change, decision-making autonomy, and career development opportunities are positively associated with positive employee evaluations of organizational change (e.g., Miller et al., 1994; Wanberg and Banas, 2000; Jimmieson et al., 2004; Armenakis and Harris, 2009; van Emmerik et al., 2009; Choi, 2011). Employees who report that they have opportunities to participate in the design and execution of change and who perceive their managers as competent and trustworthy have been shown to be more change ready and to participate more fully in change processes (Elias and Mittal, 2011). Opportunities to learn and to develop new skills through organizational change have also been shown to result in more positive employee attitudes to change (Wanous et al., 2000).

Consistent with JD-R theory (Bakker and Demerouti, 2014), Figure 1 shows change-related job resources directly influencing employee change engagement. As previously noted, both engagement and change engagement are “high-arousal” constructs, and therefore, just as job resources have been shown to lead to engagement, change-related job resources will likely lead to change engagement. Table 1 notes a number of specific change-related job resources that have been identified as positively influencing employee attitudes to change. Analogous to JD-R theory, Figure 1 also shows job-related change resources directly influencing change-related personal resources.

## Change-Related Personal Resources

Extrapolating from engagement research showing the influence of personal resources [e.g., Psychological Capital (PsyCap; Luthans et al., 2007), meaning-making, and organization-based self-esteem] on engagement (e.g., Xanthopoulou et al., 2007), it is here proposed that change-related personal resources will influence change engagement. Change-related personal resources refer to enduring psychological states, or mindsets, which shape an individual’s ability to successfully adapt to a changing work environment. Broaden and build theory (Fredrickson, 2001) suggests that the availability of change-related personal resources will serve to expand an employee’s thought and action repertoire and thereby result in their increased receptiveness to change and more active experimentation with change. In support of this reasoning, researchers have examined the relationship between PsyCap, or its constituent constructs of self-efficacy, optimism, hope, and resilience, on attitudes and reactions to change (e.g., Jimmieson et al., 2004; Hicks and Knies, 2015; Lizar et al., 2015; Kirrane et al., 2016). Jimmieson et al. (2004), for example, using a longitudinal design showed that change-related self-efficacy had a direct influence on employee attitudes to change. Furthermore, given that optimism entails viewing the environment positively and anticipating the need to successfully manage different events, it is likely that change-related optimism will also be positively associated with change engagement. Similarly, and again consistent with broaden and build theory, it is likely that individuals with higher levels of change-related hope and resilience will be more positively predisposed to change and more likely to find ways to successfully adapt to change (Rutter, 1985; Ryff and Singer, 1996; Fugate, 2013).

As per Table 1, additional change-related personal resources might include constructs such as change-related mindsets, change-related self-management, change-related self-leadership, change-related job crafting, change-related psychological safety, and change-related resilience, optimism, and hope. Personality traits such as negative affectivity, conscientiousness, openness to change, and neuroticism, which have been shown to influence employee attitudes to change (Oreg, 2003; Oreg et al., 2011; Vakola et al., 2013), are not included in Table 1 because personal resources are generally conceptualized to be more malleable and open to development than personality traits (Luthans et al., 2006).

Overall, the proposed influence of personal resources on attitudes to change reflects the widely held view that change is not possible without employees having the personal resources they need to manage and change their attitudes and behaviors (By, 2005; van den Heuvel et al., 2009). Figure 1 proposes that change-related personal resources will directly influence employee change engagement and change outcomes.

## Change-Related Job Demands

Consistent with JD-R literature that examines the influence of both job resources and job demands on individual engagement and associated outcomes, it is important to also look at the factors that may result in negative employee change experiences and that may adversely influence the extent to which they are open to, or engaged in, change. Change-related job demands refer to intellectual, physical, psychological, and social responses required by employees in a changing work environment that deplete their energy and well-being.

Beyond demands such as work pressure, role ambiguity, role conflict, job insecurity, daily hassles, and emotional demands (Albrecht et al., 2015) that form part of an employee’s everyday work experience, change-related job demands will likely become increasingly salient within the context of continuous organizational change. Employees are likely to experience increased demands during times of organizational change because they have to learn new skills, routines, and cultures and because change may disrupt existing coworker relationships and networks (van Emmerik et al., 2009). Researchers have identified workload (Simpson, 1998), uncertainty (Ashford, 1988; Bordia et al., 2004), job insecurity (Rafferty et al., 2013), role conflict (Rafferty and Griffin, 2006), role ambiguity (Smollan, 2015), and emotional demands (van Emmerik et al., 2009) as job and organizational demands that can adversely influence employee attitudes to change. Lee et al. (2017) argued that if employees experience such demands, “they are more likely to negatively respond to the change and disengage themselves from organizational change” (p. 505). Furthermore, and in line with JD-R theory, because they require sustained physical or psychological effort, change-related demands will deplete employee energy and potentially lead to adverse employee outcomes such as exhaustion, stress, and reduced well-being (Miller et al., 1994; Fein et al., 2017). Figure 1 shows change-related demands being directly associated with change-specific personal resources and change engagement. Table 1 identifies a number of change-specific demands (e.g., change-related role ambiguity, change-related job insecurity, and change-related work intensification) that may directly or indirectly impact personal resources, employee change engagement, and associated downstream organizational outcomes.

## Discussion

In this article, we have offered a model that draws from well-established research and practice. We have argued that change engagement is an important construct to be considered in future change-related research. The construct is important, given that contemporary organizations are looking for employees who, rather than being passively non-resistant to change, are energized and motivated by change and prepared to invest personal energy in the planning, implementation, review, and evaluation of change. We propose that change engagement, by virtue of its motivational and agentic qualities, provides a potentially valuable analog to the construct of employee engagement that has resonated very deeply with executives and employees in contemporary organizational contexts (Macey and Schneider, 2008).

In this article, we have argued that the proposed CEM can, after validation, parallel the JD-R model in generating interest and traction in the academic change literature and in the practice of organizational change. As with the JD-R, the proposed CEM has the advantage of being both flexible and comprehensive (Bakker and Demerouti, 2014). With respect to flexibility, although the example resources and demands identified in Table 1 are likely to be relevant to the context of ongoing organizational change, additional change-related resources and demands may be more or less salient dependent upon any specific change context under consideration. With respect to comprehensiveness, the model prompts consideration of the influence of organizational, job, and personal resources on employee attitudes to change. In this sense, the model highlights the importance of organizations adopting a “systems” approach to organizational change, whereby organizational, job, and personal resources are appropriately measured, embedded, and developed. Furthermore, as with the JD-R, the model can easily be extended to include both positive and negative attitudes to change and a range of additional organizational outcomes.

### Practical Implications

In practical terms, the proposed model can potentially be used to help organizations develop an easily communicated common language around change. Furthermore, the model or framework can potentially provide for the development of a practically useful diagnostic for organizations wanting to comprehensively understand and manage what they can do to optimize employee attitudes to change. Using the model and Table 1 as a basis for developing a survey or an interview protocol, organizations will potentially be able to collect qualitative and quantitative data about how employees are experiencing differing aspects of change and subsequently allocate and develop organizational, job, and personal resources as appropriate. The elements in Table 1 provide a mix of potential top-down and bottom-up targets for resource-boosting interventions (van den Heuvel et al., 2013) that could be helpful for managers, change agents, employees, and other related stakeholders. More generally, the model reinforces the importance of organizations creating system-level change capability (Judge and Douglas, 2009) that enables the successful implementation and adoption of ongoing organizational change. Organizations that develop change capability through change-conducive senior leadership, cultures, climates, and job-level resources are likely to have employees who are more positively and proactively predisposed to change.

### Future Research

Given the conceptual nature of the article, empirical research is needed to test and substantiate the constructs and relationships proposed. Additional research is needed to establish the construct validity of change engagement and its discriminant validity with respect to existing measures of openness to change, willingness to change, and resistance to change. Similarly, research is needed to establish the validity with respect to measures of change-related organizational resources, job resources, personal resources, and job demands.

With respect to organizational resources, Fugate (2013) noted that although “many HRM practices have been identified as drivers of organizational change—‘training, recruitment, selection, and socialization of new employees; changes in performance appraisal criteria; and incentives and rewards’ (Whelan-Berry and Somerville, 2010)—empirical research involving these practices was very limited in the past decade” (p. 187). Research is still needed to develop measures of organizational and HRM capability and to determine the strength of their association with change engagement. More research is also needed to establish the constructs of change-oriented organizational climate and culture and to examine the extent to which they are predictive of change engagement and downstream change-related and organizational outcomes.

With respect to job-relevant factors, researchers have called for further research regarding the relationships between job-level characteristics, engagement, and positive attitudes toward change (e.g., Rafferty and Griffin, 2006; van den Heuvel et al., 2017). Table 1 identifies a number of job-related change resources and demands that potentially can influence employee engagement in change. More research is needed, however, to develop defensible measures of such constructs and to determine the types of job resources and demands that have a salient influence on employee engagement in change. With respect to personal resources, Table 1 also identifies a number of personal resources that we propose will influence change engagement. Change-related psychological safety, for instance, might prove to be an important moderator or mediator of the relationships between job-related resources and employee change engagement. Change-related adaptations of existing constructs such as PsyCap (Luthans et al., 2007) might also be developed, and an assessment made of their relationships with change engagement. The moderating influence of personality factors such as dispositional openness to experience, conscientiousness, locus of control, and tolerance for ambiguity on the relationships presented in the model is also an obvious and important area for future research. Moreover, additional research across a number of change-related personal resources will help researchers and practitioners further tap into the explanatory mechanisms that, in part, account for the relationships between job-relevant job features and change engagement. We agree with Bouckenooghe (2012), who argued that there is a need for further understanding of how attitudes to change may develop and evolve differently in response to the ongoing and constant change that characterizes contemporary organizational contexts. We contend that the CEM proposed provides a potentially useful framework within which to conduct such research.

## Conclusion

Considering the increasing pace of organizational change, it is essential that researchers further develop an understanding of the factors that drive positive attitudes toward change. This is because the extent to which employees adopt or resist change has a clear impact on the amount of implementation effort, cost, and success of ongoing organizational change. In this article, we propose that the construct of change engagement is a desirable and adaptive positive psychological state and propose a model consisting of organizational, job, and personal change-related resources and demands that organizations can potentially use to self-assess the likelihood of their employees being change engaged. The model draws from engagement theory (Bakker and Demerouti, 2014) and an elaborated JD-R (Albrecht et al., 2015) to integrate constructs drawn from the change literature into a coherent and theoretically defensible framework. As previously noted, the model therefore goes some way toward addressing the lack of theoretical underpinning and integration in the research on organizational change (Oreg et al., 2011; Straatmann et al., 2016). After being subjected to validation processes, the model can potentially help organizations understand how to allocate their energies and resources to better ensure employees adopt, support, and promote ongoing organizational change. Although change engagement does not assume the mindless and uncritical acceptance of change, organizations that have well-developed and integrated change resources and capabilities will be better equipped to face the challenges and opportunities associated with ongoing organizational change.

## Author Contributions

SA was responsible for conceptualizing the constructs and the model. All authors contributed equally to the writing of the manuscript.

## Conflict of Interest

The authors declare that the research was conducted in the absence of any commercial or financial relationships that could be construed as a potential conflict of interest.
